# Monitoring of Schmallenberg virus in Spanish wild artiodactyls, 2006–2015

**DOI:** 10.1371/journal.pone.0182212

**Published:** 2017-08-16

**Authors:** Ignacio García-Bocanegra, David Cano-Terriza, Gema Vidal, Rosa Rosell, Jorge Paniagua, Saúl Jiménez-Ruiz, Carlos Expósito, Antonio Rivero-Juarez, Antonio Arenas, Joan Pujols

**Affiliations:** 1 Departamento de Sanidad Animal, Universidad de Córdoba-Agrifood Excellence International Campus (ceiA3), Córdoba, Spain; 2 Center for Animal Disease Modeling and Surveillance (CADMS), Department of Medicine & Epidemiology, School of Veterinary Medicine, University of California, Davis, California, United States of America; 3 Centre de Recerca en Sanitat Animal (CReSA)—Institut de Recerca i Tecnologia Agroalimentàries (IRTA), Campus de la Universitat Autònoma de Barcelona, Bellaterra, Barcelona, Spain; 4 Infectious Diseases Unit. Instituto Maimónides de Investigación Biomédica de Córdoba (IMIBIC), Hospital Universitario Reina Sofía de Córdoba, Universidad de Córdoba, Córdoba, Spain; Universidade de Aveiro, PORTUGAL

## Abstract

Schmallenberg disease is an emerging disease that affects domestic and wild ruminants in Europe. An epidemiological survey was carried out to assess exposure to Schmallenberg virus (SBV) in wild artiodactyls in Spain between 2006 and 2015. A total of 1751 sera from wild artiodactyls, including 1066 red deer, 304 fallow deer, 192 mouflon, 109 wild boar, 49 roe deer and 31 Spanish ibex were tested for antibodies against SBV by ELISA and confirmed by virus neutralization test. SBV was not detected between the 2006/2007 and the 2010/2011 hunting seasons. Overall seroprevalence (including samples collected between the 2011/2012 and 2014/2015 hunting seasons) was 14.6% (160/1099; 95%CI: 12.7–16.6). Mean SBV seroprevalence was 13.3±2.6% in red deer, 23.9±4.2% in fallow deer, 16.4±6.1% in mouflon and 2.8±3.1% in wild boar. No antibodies against SBV were found in roe deer or Spanish ibex. The presence of SBV RNA was confirmed in three of 255 (1.2%) spleen samples from wild ruminants analysed by rRT-PCR. In a multivariate mixed-effects logistic regression model, the main risk factors associated with SBV seroprevalence were: species (fallow deer, red deer and mouflon), age (adults) and interactions between hunting areas of more than 1000 hectares and hunting season (2012/2013, 2013/2014 and 2014/2015). The hypothesis of endemic circulation of SBV in the last few years is supported by the detection of SBV RNA in animals sampled in 2011 and 2015, as well as antibodies detected at low level in juveniles in 2012, 2013 and 2014. The results indicate that SBV circulated in wild ruminant populations in Spain during the same period when the virus was first reported in northern Europe, and at least five months before the first case was officially reported in livestock in Spain.

## Introduction

Schmallenberg virus (SBV) is an arthropod-borne *Orthobunyavirus* of the Simbu serogroup (family *Bunyaviridae*), which affects domestic and wild ruminant species. The virus is mainly transmitted by biting midges of the genus *Culicoides*. In adult animals, the syndrome is acute and non-specific. In pregnant ruminants, however, infection can lead to abortions, stillbirths and congenital malformations in newborn animals [1]. Schmallenberg virus was first reported in North Rhine-Westphalia (Germany) in summer 2011. Since then, the virus has emerged and re-emerged in livestock in various European countries. Spain reported the first outbreak of Schmallenberg disease in March 2012, in a flock of sheep in the province of Cordoba (southern Spain) [2]. Fetal malformations observed in this flock included arthrogryposis, lordosis and cerebellar hypoplasia [3].

In the last few years, serosurveys have revealed widespread exposure to SBV among wild artiodactyl species in different European countries. SBV seropositivity has been detected in red deer (*Cervus elaphus*) (range 6.0%–71.4%), fallow deer (*Dama dama*) (ranging from 0.0% to 56.3%), roe deer (*Capreolus capreolus*) (ranging from 27.3% to 80.0%), Pyrenean chamois (*Rupicapra pyrenaica*) (7.6%), European bison *(Bison bonasus*) (range 76.1%–81.8%), Alpine chamois (*R*. *rupicapra*) (4.5%), elk (*Alces alces*) (22%), European mouflon (*Ovis aries musimon*) (range 0.0%–75.0%), Alpine ibex (*Capra ibex*) (33.3%) and wild boar (*Sus scrofa*) (range 15.3%–23.4%) [2–12]. The seroprevalence levels detected in wild artiodactyls raises the question of whether these species play a role in the epidemiology of SBV, as has previously been indicated in relation to bluetongue virus [13].

The geographical distribution of different wild artiodactyl species and population densities, particularly of red deer (*Cervus elaphus*) and wild boar (*Sus scrofa*), have increased substantially in Spain in recent decades [14, 15], which has led to the frequent sharing of habitats with domestic livestock and the subsequent increase in the risk of disease transmission [16]. Even though wild artiodactyls have been suggested as a potential reservoir of SBV, information about the role of wildlife in the transmission and maintenance of SBV in Mediterranean ecosystems is still very limited. In the context of growing and expanding wild ungulate populations, we hypothesized that these species may be implicated in the epidemiology of SBV in Mediterranean ecosystems. Three hypotheses were tested: a) SBV was circulating among Spanish wild artiodactyls before the first outbreak was reported in livestock; b) wild artiodactyls may act as a natural reservoir of SBV in Spain; c) SBV is endemic in this country, even though no outbreaks have been reported in livestock in the last few years.

## Material and methods

### Ethics statement

This study did not involve purposeful killing of animals. No animals were specifically hunted for this study and ethical approval by an Institutional Animal Care and Use Committee was not deemed necessary. All samples were collected from legally hunted individuals, by authorized hunters with the correct permits and licenses and with the permission of landowners. Animals were sampled during the hunting season under Spanish and EU legislation. All collection of samples was performed following routine procedures before the design of this study, in compliance with the Ethical Principles in Animal Research. Protocols, amendments and other resources were completed according to guidelines approved by each regional autonomous government following the R.D.1337/2013 of the Ministry of Presidency of Spain (1st February 2013, BOE 8th February 2013) (https://www.boe.es/diario_boe/txt.php?id=BOE-A-2013-1337)).

### Sampling

**Fig 1:**
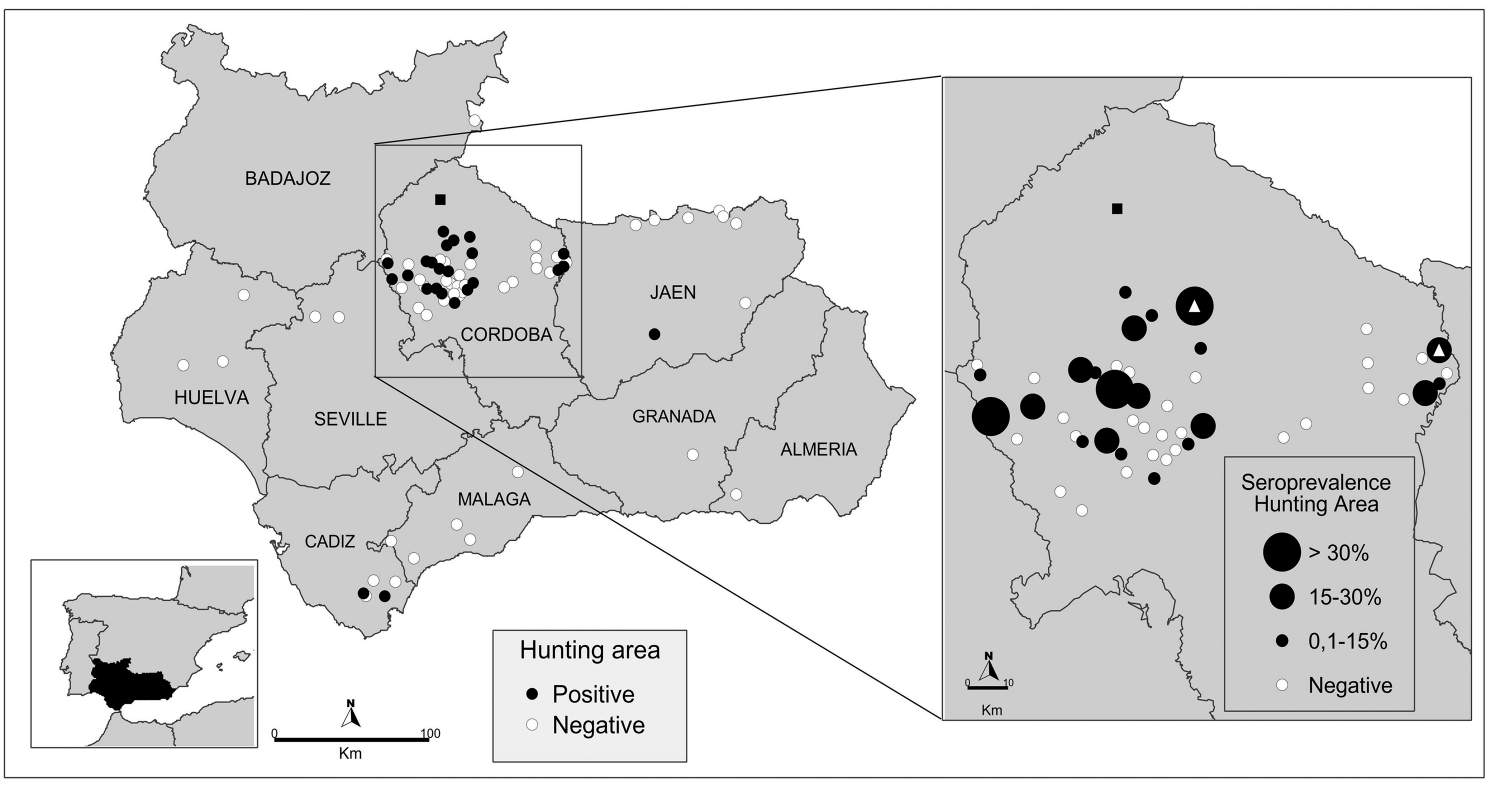
Spatial distribution of SBV in wild artiodactyls in southern Spain. Black and white dots indicate positive and negative hunting areas, respectively. White triangles indicate SBV RNA-positive animals detected. Black square indicates the geographical location of the first SBV outbreak in livestock reported in Spain.

A total of 1751 wild artiodactyls were sampled in 75 hunting areas in nine provinces located in south-central Spain (36° N—38° 60´ N, 1° 75´ W—7° 25´ W) between the hunting seasons 2006/2007 and 2014/2015 (Fig 1). The study area is characterised by a continental and Mediterranean climate, with mild winters, hot dry summers, and rainy seasons in the autumn and spring. This area presents one of the highest densities of wild artiodactyls in Spain due to intensive big game management [14, 15], with frequent sharing of habitats with livestock [17].

Blood was collected from red deer (n = 1066), fallow deer (n = 304), European mouflon (n = 192), wild boar (n = 109), roe deer (n = 49) and Spanish ibex (*Capra pyrenaica hispanica*) (n = 31). Seropositivity according to hunting season, species and province is shown in S1 Table. The animals were classified into three age groups based on tooth replacement: yearlings (< 1 year old), sub-adults (between 1 and 3 years old) and adults (> 3 years old) [18]. All samples were classified according to sex. Blood samples were taken from the thoracic cavity or by puncture of the dural venous sinuses, as previously described [19,20]. Samples were placed into sterile tubes and centrifuged at 400 g for 15 minutes. Sera were stored at -20°C until tested. In addition, spleen samples were collected at necropsy for SBV RNA detection and stored at -80°C until required for analysis.

An epidemiological questionnaire, including data on the animals sampled and hunting areas, was also completed by direct interview with gamekeepers at each hunting ground. The questionnaires were specifically designed with closed-ended questions to avoid ambiguous or lengthy answers in data collection. Epidemiological information related to the sampled animals, sampling-site features and environmental features were included in the questionnaire to obtain information about levels of exposure to potential risk factors.

### Laboratory analyses

The presence of antibodies against the SBV N protein was determined using the commercial blocking enzyme-linked immunosorbent assay (bELISA R.13.SBV.K3 INgezim Schmallenberg Compac^®^, Ingenasa, Madrid, Spain) (sensitivity 98%, specificity 99%), according to the manufacturer’s recommendations. bELISA was used for serological screening, and positive and doubtful sera by bELISA were tested by virus neutralisation test (VNT) as previously described, with minor modifications [21]. Briefly, sera were heat-inactivated and 2-fold diluted from 1:5 to 1:640. Fifty microliters of each dilution were mixed with an equal volume of EMEM containing 100 50% tissue culture infective doses (100 TCID50) of SBV (BH80/11-4, kindly provided by the Friedrich-Loeffler Institute, Germany), then incubated for one hour at 37°C. Approximately 15,000 Vero cells in 100μl of EMEM supplemented with 10% fetal calf serum were then added to each well. The plates were incubated for 3–5 days at 37°C under 5% CO_2_ atmosphere. The cells were examined by light microscopy at 3 and 6 days for the presence of SBV-specific cytopathogenic effects. To exclude individual serum toxicity, one control well without the virus was included. Positive controls (FLI-SBV 0112 positive sera) were included in each analysis. Titres were expressed as the reciprocal of the highest dilution that neutralised 100 tissue culture infective doses (100 TCID_50_) in Vero cells. Only samples that showed neutralisation (absence of cytopathic effect (CPE)) at dilutions ≥1:5 were considered positive. The cells were examined daily by light microscopy for the presence of SBV-specific cytopathogenic effects. Seropositivity was determined from samples positive by bELISA and VNT.

Testing for the presence of viral SBV RNA was performed on 255 spleen samples taken from wild artiodactyls sampled in hunting areas where at least one seropositive animal was detected. The spleen is considered a target tissue for SBV RNA detection in ruminants because viral RNA can be detected in it up to 5 weeks after infection [22]. Viral RNA was extracted directly from the spleen using a commercial kit (MagAttract^®^ 96cador^®^ Pathogen Kit QIAGEN). Samples were analysed using real-time reverse transcriptase-PCR (rRT-PCR) detecting a conserved region in the small (S) segment of the SBV genome [23]. Negative and positive (BH80/11-4) controls were included in each analysis.

### Statistical analysis

The estimated prevalence of antibodies against SBV was calculated from the ratio of positives to the total number of samples examined, using exact binomial confidence intervals (95%CI) [24]. Samples collected before the 2011/2012 hunting season, when the first seropositive animal was detected, were excluded to determine overall seroprevalence. In order to detect non-linear relationships and to standardise the scales of explanatory variables, continuous variables were categorized according to hunting management criteria for the variable “surface hunting area” (< 1000 hectares and > 1000 hectares), and using the 33 and 66 percentiles as cut-off points for the variable “distance to the nearest town” (< 5 km, 5–10 km and > 10km). Frequencies were computed and variables re-categorized on the basis of biological relevance when necessary. In order to prevent collinearity, Cramer’s V coefficient between pairs of variables was computed and those with a coefficient greater than 0.60 were considered to be correlated and were not included together in the same model. When collinear variables were detected, only the variable with the a priori stronger biological association with SBV seropositivity was retained. Pearson’s chi-square test or Fisher’s exact, when there were fewer than six observations per category, was also applied for independence of explanatory variables according to outcome.

Associations between explanatory variables and SBV seropositivity were tested by fitting a mixed-effects logistic regression model to each of the study variables, allowing different intercepts for “hunting area”. All statistically significant variables (likelihood ratio and Wald test, *P*-value < 0.10) in the bivariate analysis were selected as potential risk factors. Finally, a mixed-effects logistic regression model with different intercepts for each hunting area was fitted in order to study the effect of the variables selected on the basis of bivariate analysis. SBV seropositivity was assumed to follow a binomial distribution. For forward model building, variables were included one at a time, starting with the variable with the lowest *P*-value in bivariate analysis. If two variables correlated with each other, only the variable with the strongest statistical association with the outcome was retained. At each step, the confounding effect of the included variable was assessed by computing the change in the odds ratio. Confounding variables were those that, when added to the model, changed the OR by more than 30%, and were forced into the final model regardless of their significance level. Potential two-way interactions between all the variables were tested for significance in the model. Akaike’s Information Criterion (AIC) was used for model comparison and selection, with the lowest AIC indicating the best fit. Statistical analyses were performed using R open-source statistical software [25]. The libraries used from R statistical software were lmer4 [26], foreign [25], stats [25], vcd [27], arm [28], and tidyverse [29] as package wrapper.

## Results

A total of 179 (10.2%) of 1751 sera collected from wild artiodactyls tested positive for SBV by bELISA. Seven samples could not be analysed by the VNT due to serum cytotoxicity. Twelve sera were considered false positives because they were positive by bELISA but negative by VNT, so that the overall frequency of seropositives was 9.2% (160/1744). Between the 2006/2007 and the 2010/2011 hunting seasons, SBV antibodies were not detected in circulation. The overall seroprevalence in wild artiodactyls (including samples collected between the 2011/2012 and the 2014/2015 hunting seasons) was 14.6% (160/1099; 95%CI: 12.7–16.6). Mean SBV seroprevalence was 13.3±2.6% (87/653) in red deer, 23.9±4.2% (47/197) in fallow deer, 16.4±6.1% (23/140) in mouflon, and 2.8±3.1% (3/109) in wild boar. Seroprevalence was significantly higher in all wild ruminant species than in wild boar (*P* < 0.001).

Seropositivity was significantly higher in the province of Cordoba (16.5%; 157/952) compared to Cadiz (4.2%; 2/48; Fisher’s exact test = 5.19, *P* = 0.011) or Jaen (2.9%; 1/35; Fisher’s exact test = 4.66, *P* = 0.016), the only provinces where SBV circulation was found (Fig 1). Twenty four out of 49 (48.9%) areas sampled during the 2011/2012 hunting season presented at least one seropositive animal. Seropositivity was found between 2011/2012 and 2014/2015. Seropositive yearlings were detected during the hunting seasons of 2012/2013 (nine red deer and one mouflon in Cordoba), 2013/2014 (one fallow deer in Cadiz) and 2014/2015 (three red deer and one fallow deer in Cordoba) (Fig 1).

A total of 17 explanatory variables were considered for the bivariate analysis of SBV seropositivity in wild artiodactyl species in Cordoba province (southern Spain) (Table 1). Nine variables were finally selected from the bivariate mixed-effects model (*P* < 0.10) (Table 1). Sex was excluded from the multivariate analysis due to collinearity with the variable “species”, while “presence of fallow deer” and “presence of domestic ruminants” showed collinearity with “surface of hunting area”.

**Table 1:** Explanatory variables included in the bivariate analysis of Schmallenberg virus seropositivity in wild artiodactyl species in Cordoba province, southern Spain.

Variable	Category	Seroprevalence (%)	N° positives/total	*P-*value
	Wild boar	2.8	3/108	0.003
Species*	Red deer	15.7	86/547	
	Mouflon	17.2	23/134	
	Fallow deer	27.6	45/163	
	Yearlings	11.4	14/123	0.014
Age*	Sub-adults	16.8	52/310	
	Adults	18.5	89/481	
Sex*	Male	18.4	114/621	0.006
	Female	13.2	43/326	
	2011/2012	4.4	7/158	<0.001
Hunting season*	2012/2013	25.5	41/161	
	2013/2014	18.4	44/239	
	2014/2015	16.5	65/394	
Surface area of the hunting area (in hectares)*	< 1000	7.1	20/283	<0.001
	> 1000	20.5	137/669	
Supplementary feeding	No	7.0	7/100	0.109
	Yes	17.6	150/852	
Fenced	No	17.8	120/673	0.471
	Yes	13.3	37/279	
Restocking	No	16.4	154/939	0.365
	Yes	23.1	3/13	
Distance to the nearest town	< 5km	21.1	67/317	0.508
	5–10 km	11.3	35/310	
	>10 km	16.9	55/325	
High density of red deer*	No	4.9	5/103	0.022
	Yes	17.9	152/849	
Presence of fallow deer*	No	8.2	35/425	<0.001
	Yes	23.1	122/527	
Presence of mouflon	No	15.6	94/604	0.292
	Yes	18.1	63/348	
Presence of domestic ruminants*	No	19.1	150/786	0.001
	Yes	4.2	7/166	
Presence of rivers*	No	8.3	20/241	0.040
	Yes	19.3	137/711	
Presence of Mediterranean scrub	No	9.9	23/233	0.556
	Yes	18.6	134/719	
Presence of dehesa	No	9.4	13/138	0.781
	Yes	17.7	144/814	
Presence of pine forest	No	12.1	28/231	0.914
	Yes	17.9	129/721	

* Explanatory variables selected from the bivariate mixed-effects model (*P* < 0.10).

The multivariate mixed-effects logistic regression model (AIC of 731) showed that the main risk factors potentially associated with the individual risk of infection by SBV in wild artiodactyls were: species (fallow deer, red deer and mouflon), age (adult) and interaction between surface area of the hunting ground (>1000 hectares) and hunting season (2012/2013, 2013/2014 and 2014/2015) (Table 2). Significantly higher seropositivity was found in hunting areas of more than 1000 hectares sampled during hunting seasons 2012/2013, 2013/2014 and 2014/2015, compared to those sampled during the 2011/2012 hunting season.

**Table 2:** Results of the mixed-effects logistic regression model of risk factors associated with Schmallenberg virus seropositivity in wild artiodactyl species in Cordoba province (southern Spain).

Variable	Category	*β*	Sig.	OR	95% CI	
	2011/2012	*	*			
Hunting season	2012/2013	0.805	0.361			
	2013/2014	-0.398	0.737			
	2014/2015	0.656	0.316			
Surface hunting area	< 1000 hectares	*	*			
	> 1000 hectares	-1.255	0.150			
	Red deer	2.087	0.001	8.06	2.32	28.00
	Fallow deer	2.531	<0.001	12.56	3.20	49.31
Species	Mouflon	1.767	0.010	5.85	1.52	22.51
	Wild boar	*	*	*	*	*
Age	Yearlings	*	*	*	*	*
	Sub-adults	0.663	0.060	1.94	0.97	3.87
	Adults	1.056	0.002	2.88	1.47	5.62
Hunting season* Surface of hunting area	2011/2012*>1000 ha	*	*	*	*	*
	2012/2013*>1000 ha	3.426	0.002	30.73	3.45	273.42
	2013/2014*>1000 ha	3.201	0.016	24.76	1.78	343.24
	2014/2015*>1000 ha	1.800	0.045	6.03	1.03	35.19

* Reference category; OR. Odds ratio; 95% CI. 95% Confidence interval.

SBV RNA was detected in three out of 255 wild ruminants analysed (1.2%). The three animals positive by rRT-PCR were sampled in the province of Cordoba (Fig 1). SBV-RNA-positive animals included one adult red deer sampled in the 2011/2012 hunting season, and one sub-adult fallow deer and one yearling red deer, both from the same hunting area, sampled in the 2014/2015 hunting season.

## Discussion

Our findings confirm that wild artiodactyls were actively exposed to SBV in southern Spain during the period 2011 to 2015. Because the sample size was not geographically homogeneous, differences in seroprevalence between provinces may be associated with a certain sampling bias. Nevertheless, we detected seropositivity in three of the nine provinces analysed. Furthermore, seropositive animals were detected in 32.0% of the 75 hunting areas (48.9% of the 49 areas sampled in the 2011/2012 hunting season), which indicates widespread circulation of SBV among wild artiodactyl populations in the study area. The high seroprevalence detected in the province of Cordoba, particularly in the 2012/2013 hunting season, is in line with the first SBV outbreak among sheep in Spain in March 2012 [30].

In Spain, SBV infection and seroconversion have been reported in domestic and wild ruminant species in different regions of the country [31–34]. Our results confirm the susceptibility of red deer, fallow deer, mouflon and wild boar to SBV exposure. SBV seropositivity was determined only from samples positive by both bELISA and VNT. Several sera positive by bELISA could not be tested by VNT due to serum cytotoxicity, so that seroprevalence may have been slightly underestimated. The seroprevalence found indicates circulation of SBV in these species and is in keeping with previous reports in other European countries (Table 3). The absence of seropositivity observed in roe deer and Spanish ibex was not unexpected, given that all samples from these species were collected before the 2011/2012 hunting season when the virus was first reported in both wild and domestic ruminants. High seroprevalence, ranging between 27.3% and 80.0%, was recently detected in roe deer in different regions of Spain during the 2013–2014 period (Table 3).

**Table 3:** Prevalence of Schmallenberg virus antibodies in different wild artiodactyl species in Europe.

Species	Country	Period	No. positives/No. analysed	Analysis method	Reference
			(Seropositivity)		
Fallow deer	United Kingdom	2012	9/16 (56.3%)	ELISA	[4]
Fallow deer	Poland	2011–2012	0/16 (0.0%)	ELISA	[5]
Fallow deer	Poland	2013–2014	81/256 (22.7%)	ELISA/VNT	[6]
Fallow deer	Sweden	2012–2016	13/44 (29.5%)	ELISA/VNT	[12]
Fallow deer	Spain	2011–2015	47/197 (23.9%)	ELISA/VNT	Present study
Red deer	Italy	2007–2013	21/352 (6.0%)	ELISA/VNT	[7]
Red deer	France	2010–2012	87/486 (17.9%)	ELISA	[9]
Red deer	Poland	2010–2013	15/69 (21.7%)	ELISA	[4]
Red deer	Belgium	2011	-/- (40.5%)	ELISA	[35]
Red deer	France	2011–2014	376/983 (38.3%)	ELISA	[10]
Red deer	United Kingdom	2012	5/7 (71.4%)	ELISA	[4]
Red deer	Italy	2012–2013	21/52 (40.3%)	ELISA/VNT	[7]
Red deer	Poland	2013–2014	44/176 (30.6%)	ELISA/VNT	[6]
Red deer	Sweden	2012–2016	4/22 (18.2%)	ELISA/VNT	[12]
Red deer	Spain	2011–2015	87/653 (13.3%)	ELISA/VNT	Present study
Mouflon	Spain	2011–2013	0/75 (0.0%)	ELISA/VNT	[32]
Mouflon	Germany	2011–2014	33/44 (75%)	ELISA/VNT	[36]
Mouflon	France	2012–2014	27/73 (37.0%)	ELISA	[10]
Mouflon	Poland	2013–2014	1/71 (1.4%)	ELISA/VNT	[6]
Mouflon	Spain	2011–2015	23/140 (16.4%)	ELISA/VNT	Present study
Wild boar	Belgium	2011–2012	133/700 (19%)	-	[37]
Wild boar	Italy	2012–2013	25/107 (23.4%)	ELISA/VNT	[8]
Wild boar	Germany	2011–2014	224/1462 (15.3%)	ELISA/VNT	[36]
Wild boar	Spain	2011–2015	3/109 (2.8%)	ELISA/VNT	Present study
Roe deer	Belgium	2010–2011	97/211 (45.9%)	ELISA	[35]
Roe deer	France	2011–2014	371/746 (49.7%)	ELISA	[10]
Roe deer	Spain	2013	4/5 (80%)	ELISA/VNT	[32]
Roe deer	Spain	2013–2014	40/75 (53.3%)	ELISA	[34]
Roe deer	Sweden	2012–2016	3/11 (27.3%)	ELISA/VNT	[12]
Roe deer	Spain	2011–2015	0/49 (0.0%)	ELISA/VNT	Present study

The temporal trend in SBV seroprevalence is not homogeneous. As expected, seropositivity was not found between the 2006/2007 and 2010/2011 hunting seasons. The first seropositive animal detected was an adult red deer sampled in Cordoba province in October 2011. SBV RNA was also detected in one adult red deer sampled in November 2011. Our results confirm that SBV was circulating in wild ruminant populations in Spain at least five months before the first case was officially reported in livestock in Spain [30], which is consistent with the seropositivity detected in sheep in the same region and period [31]. Interestingly, SBV circulation was detected in wild ruminants in southern Spain in the same year that the virus was first reported in livestock in Germany [38]. Even though the movement of infected animals from northern Europe during 2011 cannot be ruled out, our results support the hypothesis of the appearance of SBV within a limited time period in different European countries.

The final multivariate mixed-effects logistic regression identified species, age, and interactions between hunting season and surface hunting as risk factors for SBV exposure in wild artiodactyls in Spain. The results showed a significantly higher SBV seropositivity in all wild ruminant species compared to wild boar. Even though antibodies against SBV have been detected in swine (Table 3), experimental infection of domestic pigs did not lead to virus replication and transmission, suggesting that suidae do not play a relevant role in the transmission of SBV [39].

The significantly higher seropositivity detected in adult animals probably reflects the greater exposure of this age group over time and the lifelong persistence of SBV antibodies. The results coincide with those previously reported in wild artiodactyl species [6,10,35]. SBV antibodies can be detected for at least 24 months post-infection in naturally infected cattle [40]. The persistence of maternal antibodies against SBV in calves is less than 6 months [40]. In our study, all seropositive yearling individuals were older than 8 months, so that antibodies detected in these animals were probably associated with active immunity, which indicates SBV circulation between the 2012/2013 and 2013/2014 hunting seasons. Seropositivity was significantly increased in hunting areas of more than 1000 hectares during the hunting seasons 2012/2013, 2013/2014 and 2014/2015 compared to the 2011/2012 hunting season. Prevalence of antibodies against SBV peaked in the 2012/2013 hunting season (25.5%), which may be due to the emergence of the virus, as well as a high percentage of susceptible animals in this period. Seroprevalence decreased during the 2013/2014 (18.4%) and 2014/2015 (16.5%) hunting seasons. Due to mortality by SBV infection has not been detected in adult ruminant species, these results may be explained by the circulation of SBV at lower level after its emergence in Spain and the incorporation of seronegative young individuals to the population during the following hunting seasons. It has been suggested that the lower seroprevalence observed in wild artiodactyls after the first SBV epidemic in livestock was associated with herd immunity [10]. Further longitudinal studies are needed to assess with more detail the effect of the hunting season in the temporal dynamic of SBV in the wild ungulate populations in Spain.

The presence of SBV RNA in red deer and fallow deer confirms the susceptibility of red deer and fallow deer to SBV infection. To the best of our knowledge, this is the first time SBV RNA has been detected in these species. The low frequency of SBV RNA-positive animals is consistent with the short duration of viraemia detected in domestic ruminants [22,43,^41^]. The presence of SBV RNA-positive animals confirms the circulation of the virus during the years 2011 and 2015.

In conclusion, the results obtained indicate that wild artiodactyls may act as potential natural reservoirs of SBV in Spain. The detection of antibodies against SBV during the 2011/2012 to 2014/2015 hunting seasons and the presence of seropositivity in juvenile animals during the 2012/2013 and 2013/2014 hunting seasons suggest uninterrupted, endemic circulation of SBV in southern Spain between 2011 and 2015. Seroprevalence level detected in young animals, suggest that SBV has circulated at low level in Spain since its emergence in 2012. Because wild and domestic ruminants in the studied region frequently share the same habitats, continuous transmission of SBV among wild ruminants may increase the risk of spillback transmission to livestock [10]. Serological and virological results indicate that SBV was circulating in wild ruminant populations in Spain in the same period when the virus was first reported in livestock in Germany, and months before the first outbreak was confirmed in Spain. Serosurveillance for wild artiodactyls, particularly yearling fallow deer and red deer, would be a useful tool for detection of SBV circulation, especially in areas where vaccination programs have been implemented in livestock.

## Supporting information

S1 Table Average SBV seropositivity according to hunting season, species and province. (DOC)

## References

[1] EFSA, 2014. European Food Safety Authority. Schmallenberg virus: State of Art. EFSA Journal 2014; 12: 3681.

[2] EFSA, (European Food Safety Authority), 2012. Schmallenberg virus: analysis of the epidemiological data. EFSA Journal 2012; 10: 2768.

[3] Beer M, Conraths FJ, Van der poel WHM. Schmallenberg virus—a novel orthobunyavirus emerging in Europe. Epidemiol Infect 2013; 141: 1–8. doi: 10.1017/S0950268812002245 23046921 10.1017/S0950268812002245 PMC9152057

[4] Barlow A, Green P, Banham T, Healy N. Serological confirmation of SBV infection in wild British deer. Vet Rec 2013; 172: 429 doi: 10.1136/vr.f2438 23603727 10.1136/vr.f2438

[5] Larska M, Krzysiak M, Smreczak M, Polak MP, Zmudzinski JF. First detection of Schmallenberg virus in elk (*Alces alces*) indicating infection of wildlife in Białowieza National Park in Poland. Vet J 2013; 198: 279–281. doi: 10.1016/j.tvjl.2013.08.013 24021421 10.1016/j.tvjl.2013.08.013

[6] Larska M, Krzysiak M, Kęsik-Maliszewska J, Rola J. Cross-sectional study of Schmallenberg virus seroprevalence in wild ruminants in Poland at the end of the vector season of 2014. BMC Vet Res 2014; 10: 967 doi: 10.1186/s12917-014-0307-3 25528665 10.1186/s12917-014-0307-3 PMC4299547

[7] Chiari M, Sozzi E, Zanoni M, Alborali LG, Lavazza A, Cordioli P. Serosurvey for Schmallenberg virus in alpine wild ungulates. Transbound Emerg Dis 2014a; 61: 1–3. 10.1111/tbed.12158 24034277

[8] Chiari M, Lelli D, Sozzi E, Moreno A, Alborali L, Zanoni M, et al. Serosurveillance for Schmallenberg virus (SBV) in wild boar (Sus scrofa) in Northern Italy. Proceedings of the EWDA conference 2014b; August 25–29, Edinburgh, UK.

[9] Laloy E, Bréard E, Sailleau C, Viarouge C, Desprat A, Zientara S, et al Schmallenberg virus infection among red deer, France, 2010–2012. Emerg Infect Dis 2014; 20: 131–134. doi: 10.3201/eid2001.130411 24377838 10.3201/eid2001.130411 PMC3884713

[10] Rossi S, Viarouge C, Faure E, Gilot-Fromont E, Gache K, Gibert P, et al. Exposure of Wildlife to the Schmallenberg Virus in France (2011–2014): Higher, Faster, Stronger (than Bluetongue)! Transbound Emerg Dis 2015. doi: 10.1111/tbed.12371. 10.1111/tbed.12371 25958882

[11] Tavernier P, Sys S, De Clercq K, De Leeuw I, Caij A, De Baere M, et al Serologic screening for 13 infectious agents in roe deer (*Capreolus capreolus*) in Flanders. Infect Ecol Epidemiol 2015; 5. 10.3402/iee.v5.29862 PMC4660936 26609692

[12] Malmsten A, Malmsten J, Blomqvist G, Näslund K, Vernersson C, Hägglund S, et al Serological testing of Schmallenberg virus in Swedish wild cervids from 2012 to 2016. BMC Vet Res 2017; 13(1):84 doi: 10.1186/s12917-017-1005-8 28376790 10.1186/s12917-017-1005-8 PMC5379663

[13] Lorca-Oró C, López-Olvera JR, Ruiz-Fons F, Acevedo P, García-Bocanegra I, Oleaga Á, et al Long-term dynamics of bluetongue virus in wild ruminants: relationship with outbreaks in livestock in Spain, 2006–2011. PLoS One 2014; 9(6):e100027. 10.1371/journal.pone.0100027 PMC4062458 24940879

[14] Acevedo P, Ruiz-Fons F, Vicente J, Reyes-Garcia R, Alzaga V, Gortazar C. Estimating red deer abundance in a wide range of management situations in Mediterranean habitats. J Zool 2008; 276: 37–47.

[15] Bosch J, Peris S, Fonseca C, Martinez M, De la Torre A, Iglesias I, et al Distribution, abundance and density of the wild boar on the Iberian Peninsula, based on the CORINE program and hunting statistics. Folia Zool 2012; 61: 138–51.

[16] Gortázar C, Acevedo P, Ruiz-Fons F, Vicente J. Disease risks and overabundance of game species. Eur J Wildl Res 2006; 52: 81–87.

[17] Kukielka E, Barasona JA, Cowie CE, Drewe JA, Gortazar C, Cotarelo I, et al Spatial and temporal interactions between livestock and wildlife in South Central Spain assessed by camera traps. Prev Vet Med 2013; 112: 213–221. doi: 10.1016/j.prevetmed.2013.08.008 24050782 10.1016/j.prevetmed.2013.08.008

[18] Sáenz de Buruaga M, Lucio-Calero A, Purroy-Iraizoz FJ. Reconocimiento de sexo y edad en especies cinegéticas, 1st ed. Edilesa, Spain; 2001.

[19] Arenas‐Montes A, García‐Bocanegra I, Paniagua J, Franco JJ, Miró F, Fernández‐Morente M, et al Blood sampling by puncture in the cavernous sinus from hunted wild boar. Eur J Wildl Res 2013; 59: 299‐303.

[20] Jiménez-Ruiz S, Arenas-Montes A, Cano-Terriza D, Paniagua J, Pujols J, Miró F, et al Blood extraction method by endocranial venous sinuses puncture in hunted wild ruminants. Eur J Wildl Res 2016; 62: 775–778.

[21] Loeffen W, Quak S, de Boer-Luijtze E, Hulst M, van der Poel W, Bouwstra R, et al Development of a virus neutralisation test to detect antibodies against Schmallenberg virus and serological results in suspect and infected herds. Acta Vet Scand 2012; 54: 44 doi: 10.1186/1751-0147-54-44 22871162 10.1186/1751-0147-54-44 PMC3503834

[22] Wernike K, Eschbaumer M, Schirrmeier H, Blohm U, Breithaupt A, Hoffmann B, et al Oral exposure, reinfection and cellular immunity to Schmallenberg virus in cattle. Vet Microbiol 2013; 165:155–159. doi: 10.1016/j.vetmic.2013.01.040 23452751 10.1016/j.vetmic.2013.01.040

[23] Bilk S, Schulze C, Fischer M, Beer M, Hlinak A, Hoffmann B. Organ distribution of Schmallenberg virus RNA in malformed newborns. Vet Microbiol. 2012; 159: 236–238. doi: 10.1016/j.vetmic.2012.03.035 22516190 10.1016/j.vetmic.2012.03.035

[24] Martin S, Meek A, Willeberg P. Veterinary epidemiology: principles and methods. Ames, Iowa: Iowa State University Press; 1987.

[25] R Core Team (2016). foreign: Read Data Stored by Minitab, S, SAS, SPSS, Stata, Systat, Weka, dBase, R package version 0.8–67. https://CRAN.R-project.org/package=foreign.

[26] Bates D, Maechler M, Bolker B, Walker S. Fitting Linear Mixed-Effects Models Using lme4. J Stat Softw 2015; 67:1–48.

[27] Meyer D, Zeileis A, Hornik K. vcd: Visualizing Categorical Data. The Strucplot Framework: Visualizing Multi-Way Contingency Tables with vcd. J Stat Softw 2006; 67(3):1–48.

[28] Gelman A, Su YS (2016). arm: Data Analysis Using Regression and Multilevel/Hierarchical Models. R package version 1.9–3. https://CRAN.R-project.org/package=arm.

[29] Wickham H (2016). tidyverse: Easily install and load “Tidyverse” packages. R package version 1.0.0, https://CRAN.R-project.org/package=tidyverse.

[30] RASVE, 2012. Red de Alerta Sanitaria Veterinaria. Ministerio de Medio Ambiente Medio Rural y Marino (MAPYA). Available from: http://rasve.magrama.es/RASVE_2008/Publica/Focos/Consulta.aspx.

[31] Astorga RJ, Reguillo L, Hernández LM, Cardoso-Toset F, Tarradas C, Maldonado A, et al Serosurvey on Schmallenberg virus and selected ovine reproductive pathogens in culled ewes from Southern Spain. Transbound Emerg Dis 2014; 61: 4–11. doi: 10.1111/tbed.12188 24219149 10.1111/tbed.12188

[32] Fernández-Aguilar X, Pujols J, Velarde R, Rosell R, López-Olvera JR, Marco I, et al Schmallenberg Virus Circulation in High Mountain Ecosystem, Spain. Emerg Infect Dis 2014; 20: 1062–1064. doi: 10.3201/eid2006.130961 24857166 10.3201/eid2006.130961 PMC4036760

[33] Balseiro A, Royo LJ, Gómez Antona A, García Marín JF. First confirmation of Schmallenberg Virus in cattle in Spain: Tissue distribution and pathology. Transbound Emerg Dis 2015; 62: 62–65. 10.1111/tbed.12185 24191854

[34] Díaz JM, Prieto A, López C, Díaz P, Pérez A, Panadero R, et al High spread of Schmallenberg virus among roe deer (*Capreolus capreolus*) in Spain. Res Vet Sci 2015; 102: 231–233. doi: 10.1016/j.rvsc.2015.09.001 26412550 10.1016/j.rvsc.2015.09.001

[35] Linden A, Desmecht D, Volpe R, Wirtgen M, Gregoire F, Pirson J, et al Epizootic spread of Schmallenberg virus among wild cervids, Belgium, Fall 2011. Emerg Infect Dis 2012; 18: 2006–2008. doi: 10.3201/eid1812.121067 23171763 10.3201/eid1812.121067 PMC3557893

[36] Mouchantat S, Wernike K, Lutz W, Hoffmann B, Ulrich RG, Börner K, et al A broad spectrum screening of Schmallenberg virus antibodies in wildlife animals in Germany. Vet Res 2015; 46: 99 doi: 10.1186/s13567-015-0232-x 26394618 10.1186/s13567-015-0232-x PMC4579581

[37] Desmecht D, Garigliany MM, Beer M, Schirrmeier H, Paternostre J, Volpe R, et al. Detection of antibodies against Schmallenberg virus in wild boars, Belgium, 2010–2012. Proceedings of the UIGB congress 2013; August 27–29, Brussels, Belgium.

[38] Hoffmann B, Scheuch M, Höper D, Jungblut R, Holsteg M, Schirrmeier H, et al Novel Orthobunyavirus in Cattle, Europe 2011. Emerg Infect Dis 2012; 18: 469–472. doi: 10.3201/eid1803.111905 22376991 10.3201/eid1803.111905 PMC3309600

[39] Poskin A, Van Campe W, Mostin L, Cay B, De Regge N. Experimental Schmallenberg virus infection of pigs. Vet Microbiol 2014; 170: 398–402. doi: 10.1016/j.vetmic.2014.02.026 24679959 10.1016/j.vetmic.2014.02.026

[40] Elbers ARW, Stockhofe-Zurwieden N, van der Poel WHM. Schmallenberg virus antibody persistence in adult cattle after natural infection and decay of maternal antibodies in calves. Vet Res 2014; 10: 103. 10.1186/1746-6148-10-103 PMC4013805 24885026

[41] Martinelle L, Poskin A, Dal Pozzo F, Mostin L, Van Campe W, Cay AB, et al Three Different Routes of Inoculation for Experimental Infection with Schmallenberg Virus in Sheep. Transbound Emerg Dis 2015 doi: 10.1111/tbed.12356 25891033 10.1111/tbed.12356

