# Supplementary material for: Monitoring of Schmallenberg virus in Spanish wild artiodactyls, 2006–2015
Source: PLoS One. 2017 Aug 16;12(8):e0182212. doi: 10.1371/journal.pone.0182212 (PMC5559100; doi:10.1371/journal.pone.0182212)
Supplement: S1 Table — (DOC) [file pone.0182212.s001.doc]

| Province | Hunting season | Species | | | | | | TOTAL |
| --- | --- | --- | --- | --- | --- | --- | --- | --- |
|  |  | Spanish Ibex | Red deer | Roe deer | Fallow deer | Wild boar | Mouflon |  |
| Córdoba | 2008-2009 |  | 0/13 (0.0%) |  | 0/2 (0.0%) |  | 0/3 (0.0%) | 0/18 (0.0%) |
|  | 2009-2010 |  | 0/83 (0.0%) |  | 0/42 (0.0%) |  | 0/13 (0.0%) | 0/138 (0.0%) |
|  | 2010-2011 |  | 0/75 (0.0%) |  | 0/4 (0.0%) |  |  | 0/79 (0.0%) |
|  | 2011-2012 |  | 7/106 (6.6%) |  | 0/42 (0.0%) |  | 0/11 (0.0%) | 6/159 (3.8%) |
|  | 2012-2013 |  | 29/78 (37.2%) |  | 0/3 (0.0%) | 2/53 (3.8%) | 10/27 (37.0%) | 41/161 (25.5 %) |
|  | 2013-2014 |  | 6/93 (6.5%) |  | 28/48 (58.3%) | 1/55 (1.8%) | 9/45 (20.0%) | 44/241 (18.3%) |
|  | 2014-2015 |  | 44/274 (16.1%) |  | 17/72 (23.6%) |  | 4/51 (7.8%) | 65/397 (16.4) |
|  | Total |  | 86/722 (11.9%) |  | 45/213 (21.1%) | 3/108 (2.8%) | 23/150 (15.3%) | 156/1193 (13.1%) |
| Cádiz | 2007-2008 |  | 0/3 (0.0%) | 0/1 (0.0%) | 0/7 (0.0%) |  |  | 0/11 (0.0%) |
|  | 2008-2009 |  | 0/4 (0.0%) | 0/18 (0.0%) |  |  |  | 0/22 (0.0%) |
|  | 2009-2010 |  | 0/5 (0.0%) | 0/27 (0.0%) | 0/20 (0.0%) |  | 0/10 (0.0%) | 0/62 (0.0%) |
|  | 2010-2011 |  | 0/7 (0.0%) | 0/2 (0.0%) | 0/24 (0.0%) |  | 0/15 (0.0%) | 0/48 (0.0%) |
|  | 2011-2012 |  | 0/8 (0.0%) |  | 0/14 (0.0%) |  | 0/2 (0.0%) | 0/24 (0.0%) |
|  | 2012-2013 |  | 0/14 (0.0%) |  | 1/2 (50.0%) |  |  | 1/16 (6.3%) |
|  | 2013-2014 |  | 1/9 (11.1%) |  |  |  |  | 1/9 (11.1%) |
|  | Total |  | 1/50 (2.0%) | 0/48 (0.0%) | 1/67 (1.5%) |  | 0/27 (0.0%) | 2/192 (1.0%) |
| Jaén | 2006-2007 |  | 0/18 (0.0%) |  | 0/5 (0.0%) |  | 0/2 (0.0%) | 0/25 (0.0%) |
|  | 2007-2008 | 0/1 (0.0%) | 0/31 (0.0%) |  |  |  |  | 0/32 (0.0%) |
|  | 2008-2009 |  | 0/22 (0.0%) |  |  |  | 0/4 (0.0%) | 0/26 (0.0%) |
|  | 2009-2010 | 0/8 (0.0%) | 0/7 (0.0%) |  |  |  |  | 0/15 (0.0%) |
|  | 2010-2011 |  | 0/47 (0.0%) |  | 0/1 (0.0%) |  |  | 0/48 (0.0%) |
|  | 2014-2015 |  | 0/13 (0.0%) |  | 1/18 (5.6%) |  | 0/4 (0.0%) | 1/35 (2.9%) |
|  | Total | 0/9 (0.0%) | 0/138 (0.0%) |  | 1/24 (4.2%) |  | 0/10 (0.0%) | 1/181 (0.6%) |
| Málaga | 2007-2008 | 0/8 (0.0%) | 0/5 (0.0%) |  |  |  |  | 0/13 (0.0%) |
|  | 2008-2009 | 0/3 (0.0%) | 0/46 (0.0%) | 0/1 (0.0%) |  |  |  | 0/50 (0.0%) |
|  | 2009-2010 | 0/2 (0.0%) |  |  |  |  | 0/4 (0.0%) | 0/6 (0.0%) |
|  | 2013-2014 | 0/2 (0.0%) |  |  |  |  |  | 0/2 (0.0%) |
|  | Total | 0/15 (0.0%) | 0/51 (0.0%) | 0/1 (0.0%) |  |  | 0/4 (0.0%) | 0/71 (0.0%) |
| Seville | 2006-2007 |  | 0/8 (0.0%) |  |  |  |  | 0/8 (0.0%) |
|  | 2007-2008 |  | 0/3 (0.0%) |  |  |  |  | 0/3 (0.0%) |
|  | 2008-2009 |  | 0/6 (0.0%) |  |  |  |  | 0/6 (0.0%) |
|  | 2009-2010 |  | 0/14 (0.0%) |  |  |  |  | 0/14 (0.0%) |
|  | 2010-2011 |  | 0/1 (0.0%) |  |  |  |  | 0/1 (0.0%) |
|  | 2011-2012 |  | 0/4 (0.0%) |  |  |  |  | 0/4 (0.0%) |
|  | 2013-2014 |  | 0/2 (0.0%) |  |  | 0/1 (0.0%) |  | 0/3 (0.0%) |
|  | 2014-2015 |  | 0/16 (0.0%) |  |  |  |  | 0/16 (0.0%) |
|  | Total |  | 0/54 (0.0%) |  |  | 0/1 (0.0%) |  | 0/55 (0.0%) |
| Huelva | 2008-2009 |  | 0/1 (0.0%) |  |  |  | 0/1 (0.0%) | 0/2 (0.0%) |
|  | 2009-2010 |  | 0/9 (0.0%) |  |  |  |  | 0/9 (0.0%) |
|  | 2011-2012 |  | 0/28 (0.0%) |  |  |  |  | 0/28 (0.0%) |
|  | Total |  | 0/38 (0.0%) |  |  |  | 0/1 (0.0%) | 0/39 (0.0%) |
| Badajoz | 2013-2014 |  | 0/13 (0.0%) |  |  |  |  | 0/13 (0.0%) |
|  | Total |  | 0/13 (0.0%) |  |  |  |  | 0/13 (0.0%) |
| Granada | 2008-2009 | 0/4 (0.0%) |  |  |  |  |  | 0/4 (0.0%) |
|  | Total | 0/4 (0.0%) |  |  |  |  |  | 0/4 (0.0%) |
| Almería | 2009-2010 | 0/3 (0.0%) |  |  |  |  |  | 0/3 (0.0%) |
|  | Total | 0/3 (0.0%) |  |  |  |  |  | 0/3 (0.0%) |
| Total | | 0/31 (0.0%) | 87/1066 (8.2%) | 0/49 (0.0%) | 47/304 (15.5%) | 3/109 (2.8%) | 23/192 (12.0%) | 159/1751 (9.1%) |
